# Mr. Fogo on Chronic Hepatitis

**Published:** 1812-08

**Authors:** A. Fogo

**Affiliations:** Newcastle


*To the Editors of the Medical and Physical Journal.*

GENTLEMEN,

IN No. 158 of the Journal, there is a case written by Dr. Thompson. The patient complained of a "dull pain, or rather a sense of uneasiness, in the region of the liver, attended attended with a short dry cough, alternations of heat and cold, quick soft pulse, diarrhœa, and much emaciation, nausea, stinking taste in his mouth, sometimes threw up a great gush of corruption, had been declining in health, with disordered stomach, slight pains in the side for many months; but the vomiting had only continued a few weeks."

The above is evidently a genuine case of chronic hepatitis, allowed, from bad or no treatment, to arrive at suppuration, and which fortunately broke into the stomach. Whether the patient took any medical advice or medicines during "the many months," is not declared. The case from that omission is imperfect; but, when it is considered that the disease is not understood by many of the profession, it is of less consequence, as he would have got no medicines necessary to remove the obstruction of the biliary or other vessels in the liver, in the early stage of the disease.

Dr. Thompson may perhaps be surprised at the name of chronic hepatitis; for, if he has acquired no more knowledge of it than he received from the lectures and writings of our late venerable professor, he will know very little about it, except the name.

Dr. Cullen said there were two kinds of hepatitis, the acute and the chronic. "The chronic hepatitis very often does not exhibit any symptoms of pyrexia, and is only discovered to have happened by our finding in the liver, upon dissection, large abscesses, which were presumed to be the effect of some previous inflammation. As this chronic inflammation is seldom to be certainly known, and therefore does not lead to any determined practice, we omit treating of it here." His learned annotator, Dr. Rotherham, (F. L. 414,) has lulled the profession still more into a fatal blindness, ignorance, and security, by telling them, "It is doubtful whether this chronic hepatitis ever exists." Such must be the ignorance under which thousands of Dr. Cullen's hearers and readers labor, if they have not discovered the obscure disease by their own experience in practice. Dr. Cullen had read many books, but not having met with any regular description of the disease, nor ever being able to discover it before death, he could not interweave it into his lectures or writings, as he did other diseases. This proves that there is no information to be got in any book, either ancient or modern. This also shows that there is a great chasm in the system of physic; that the largest organ in the body, and of the most essential use and importance, should have been, and still is, forgot; the functions and diseases, either acute or chronic, of such an indispensible organ, ought to be as well known as those of any more insignificant gland,

Dr.

Dr. Thompson, repeating Dr. Cullen's sentiments, says, "The liver is certainly an organ possessing less sensibility than most others, and hence its diseases, being frequently unmarked by any very striking symptoms, may easily escape detection; or, if detected, may appear too trifling to demand that active practice which ought to be adopted." Again: "Hepatic complaints are, I think, more obscure, and more frequently mistaken than those of any other organ in the human body." There are as many symptoms which point out the slightest diseases of the liver, as those belonging to any of the other internal organs, if the practitioner will attend to them; but he must not expect any assistance from lectures or books. These considerations ought to incline us to double our diligence, to enable us to detect the slightest symptom of disease in an organ of such importance to the health and life of the patient. The chronic diseases of that gland are not only overlooked, but the gland itself is scarcely thought of; and, in the apparent sentiments of both ancients and moderns, it seems of no more consideration, than a large body hung up at the top of the abdominal viscera for the purpose of filling up the cavity.

Dr. Thompson regrets that we have no "systematic arrangement of the diseases of the liver, and of the symptoms by which they may be known and distinguished." The anatomical structure of the liver is as well known as that of any other internal or external gland, and sufficient for practical purposes. Our knowledge of the manner in which secretion is performed in the various glands is very contracted, and must continue to be so; but, if we discover that any gland is not secreting its proper fluid and in sufficient quantity, we must consider the gland as in a diseased state, and endeavor to restore it to a healthy state. The knowledge of the nature and functions of the very important organ—the liver, the profession is in possession of, is very small: but who is likely or capable to increase it, while it is so generally forgot and neglected? Whoever attempts to write, he will produce an original essay *indictum ore alio;* he will not be able to swell his book with quotations from the ancients or moderns. If he has had much experience in the treatment of the diseases of that organ, and takes a comprehensive view of its effects when in a diseased state, he will include several that are now called idiopathic diseases, but which never were, or ever can be, more than effects and symptoms of the obstructed state of the circulation through the various vessels of the liver, constituting a disease of the name of chronic hepatitis.

In the concluding part of his paper, Dr. Thompson vacillates between two opinions. He says, "The great disproportion between its apparent uses (the bile) and the vast preparation made to procure it, have led me to a suspicion that the principal purpose of the liver is not the secretion of bile, but that it separates something noxious from the blood, &c." I refer the reader to his paper to save transcribing. There is no disproportion between its apparent uses and the vast preparation made to procure it. Its indispensible use, (which Dr. Thompson does not appreciate sufficiently high,) is in exact proportion to the vast preparation to procure it, nor could it have been procured without that vast preparation. He next says, "That the bile does answer *some useful* purposes; expatiates on its real uses in the system, &c." His allowing that it answers some useful purposes, is but a meagre acknowledgment of its great and indispensible use, and seems an opinion rather extorted; and his comparing the functions of the liver to those of the kidneys is a great degradation. It appears also, that although Dr. Thompson mentions some of the symptoms by which the disease may be discovered in its early stage, he has not chose to give it a name. That there was an abscess somewhere, was evident from the fœtid purulent matter brought up by vomiting. Dr. Thompson may not have met with many patients with chronic hepatitis; perhaps the disease is not common in his circle, or perhaps it is not discovered till after death, as Dr. Cullen tells us; or, as in the present case, before death, when from want of proper treatment the chronic inflammation has been allowed, or rather forced into suppuration by improper medicines.

Dr. Thompson will, I hope, excuse me for animadverting so freely on his paper; as he says, he has "formed only mere hypothetical conjectures," what I have said may possibly have the effect of drawing his attention to an organ, and to a class of diseases which stand in much need of further elucidation. In the above case, Dr. Thompson was excusable in not preventing suppuration, as the abscess was opened before he saw the patient; but I doubt, from Dr. Thompson's vacillating opinion, and forming mere hypothetical conjectures, if he had been consulted through the "many months" previous to the rupture, he might not have discovered the disease in its early stage, and prevented suppuration by proper treatment.

I think myself justified in saying so much, from observing the great want of attention to this disease by three medical practitioners who attended the patient Dr. Thompson refers to. The case is in the Medical and Physical Journal, No. 156. I will take the liberty to make some remarks on the treatment.

Mr.

Mr. Paxton was first called on the 8th of November, 1787. Thought the patient under strong symptoms of an autumnal intermittent, which was said to be nearly removed in three or four days by emetics, laxatives, and antimonials. The type of the intermittent is not mentioned, or whether regular or not. On the 13th, only five days after, the patient was worse, and sent for a physician, who, prescribing neutrals and evacuants, pronounced him free from danger. Some small degree of fever still subsisted, and he complained of an "obtuse or dull pain on the right side, near the ends of the false ribs." In this state he continued to the end of December, a space of forty-six days. During all this time the obtuse dull pain was never attended to, which is a proof that the real disease was overlooked; and, instead of attempting to remove the obstruction, the patient was ordered an emetic and purges, under the supposition that the disease was intermittent. It was natural for these three practitioners to neglect the pain in the side, from never having heard that such a disease as chronic hepatitis ever existed.

The late Dr. Gregory, in his lectures, told us, that the liver was more subject to obstructions (for very obvious reasons) than any other organ in the system. Dr. Cullen, who succeeded him in the practical chair, never could discover the disease till he saw its effects on dissection. Dr. Rotherham doubted that there ever was such a disease. Dr. Darwin copies Dr. Cullen's few random symptoms, and leaves the affair as he found it. Where then is information to be got?

On the 29th of December the patient had, what he thought, a severe fit of ague. It was more probable to be the effects of suppuration having begun in the liver. This was also overlooked, as there was no return. He took a great variety of medicines, neutrals, bark, bitters, &c. not very proper for the disease, but rather the reverse, till the 24th of January, when a seton was made on the hepatic region. (Query?) On the evening an abscess burst, which they never expected to have existed. So far from suspecting such a termination, two of the party "could not assent to its being a rupture of an abscess in the liver." Such was the want of attention, or rather knowledge, of the uses and functions of that important organ, or of its diseases, that these three practitioners attended a man seventy days, without discovering, or even suspecting, any disease in the biliary system; and the rupture so unexpected occasioned great surprise.

Dr. Thompson's case was serious; but Paxton's may be compared to a volcano, pouring out its contents during five

 months, months, which, as per account current, amounted to more than eighty pounds of purulent offensive matter!!!

These are the unblessed effects of our teachers and writers, and all before them, never having paid any attention to that most important organ in the system; nor having it in their power to direct the attention of their innumerable hearers and readers to that obscure disease.

These two cases, had there never been another, ought to be an awful warning to the profession, not to overlook or neglect an obtuse dull pain on the right side, near the ends of the false ribs. There an organ is placed of indispensible use in the digestion, chylification, &c. The disease is very common in this neighbourhood; a week seldom passes without one or more patients applying for advice; and in many hundred cases I have been able to detect the obscure disease in its early stage, and been fortunate to remove the obstruction before it arrived at suppuration, or formed any other disease. But, while practitioners give the symptoms of chronic hepatitis the names of stomach complaints, nervous or hysterical, I fear little progress will be made in the discovery of the real disease.

I am, &c.

*Newcastle, June* 10, 1812. A. FOGO.

---

*To the Editors of the Medical and Physical Journal.*

